# Research Progress of Genetic Structure, Pathogenic Mechanism, Clinical Characteristics, and Potential Treatments of Coronavirus Disease 2019

**DOI:** 10.3389/fphar.2020.01327

**Published:** 2020-08-27

**Authors:** Chunsheng Zhu, Bao Sun, Xiaochuan Zhang, Bing Zhang

**Affiliations:** ^1^Department of Chinese Medicine, The First Affiliated Hospital of Zhengzhou University, Zhengzhou, China; ^2^Department of Pharmacy, The Second Xiangya Hospital, Central South University, Changsha, China; ^3^Department of Clinical Chinese Pharmacy, School of Chinese Materia Medica, Beijing University of Chinese Medicine, Beijing, China

**Keywords:** angiotensin converting enzyme 2, coronavirus disease 2019, cytokine storm, severe acute respiratory syndrome coronavirus-2, pathogenic mechanism

## Abstract

Coronavirus disease 2019 (COVID-19) is a global pandemic infectious disease caused by severe acute respiratory syndrome coronavirus-2 (SARS-CoV-2), and currently affects more than 8 million people worldwide. SARS-CoV-2 mainly invades the cells by binding to the angiotensin converting enzyme 2 (ACE2) receptor, leading to the injury of respiratory system, cardiovascular system, digestive system, and urinary system, and even secondary to acute respiratory distress syndrome (ARDS) and systemic inflammatory response, resulting in multiple organ failure. In this review, mainly focusing on biogenesis and pathogenic mechanisms, we describe the recent progress in our understanding of SARS-CoV-2 and then summarize and discuss its crucial clinical characteristics and potential mechanism in different systems. Additionally, we discuss the potential treatments for COVID-19, aiming at a better understanding of the pathogenesis of SARS-CoV-2 and providing new ideas for the personalized treatment of COVID-19.

## Introduction

In December 2019, there was an outbreak of unidentified pneumonia due to the novel coronavirus in China, and when the virus was first isolated from pneumonia cases it was named 2019 novel coronavirus (2019-nCoV) (Ludwig and Zarbock, 2020; Wu F. et al., 2020). As more information and genetic analyses became available, the virus was denominated severe acute respiratory syndrome coronavirus 2 (SARS-CoV-2) by International Committee on Taxonomy of Virus (Mackenzie and Smith, 2020). Meanwhile, the 2019-nCoV was subsequently renamed coronavirus disease (COVID-19) by the World Health Organization (WHO) (Qiu H. et al., 2020). Although a large number of measures have been taken to control the disease, the emerging SARS-CoV-2 has spread globally and poses tremendous threat to global public health. On March 11th, 2020, SARS-CoV-2 was declared a pandemic by the WHO, and later the sixth public health emergency of international concern (Sarzi-Puttini et al., 2020). As of August 3th, 2020, more than 17,918,582 confirmed cases including more than 686,703 deaths have been reported worldwide, affecting at least 200 countries or territories (https://www.who.int/emergencies/diseases/novelcoronavirus-2019/situation-reports (2020)).

Currently, the transmission of pneumonia associated with SARS-CoV-2 has not yet been eliminated, therefore, it is important to identify the origin, local host, and evolution pathway of SARS-CoV-2 to prevent further transmission. Phylogenetic analysis shows that SARS-CoV-2 is a new member of the Coronaviridae family, which is closely related (approximately 88%) to two bat-derived SARS-like coronaviruses, bat-SL-CoVZC45, and bat-SL-CoVZXC21. However, it is distinct from SARS-CoV (approximately 79%) and Middle East respiratory syndrome coronavirus (approximately 50%) (Lu et al., 2020). Of note, SARS-CoV-2 is 96.2–96.3% identical with bat coronavirus RaTG13 from bats in Yunnan province at the whole-genome level (Paraskevis et al., 2020; Zhou et al., 2020), providing its probable origin from bats. Interestingly, previous findings suggested that snake and turtles could be the potential expanded hosts of SARS-CoV-2 (Liu et al., 2020b; Tiwari et al., 2020). More recently, a study by Zhang et al. showed that pangolin-CoV was 91.02 and 90.55% identical to SARS-CoV-2 and bat-CoV RaTG13 throughout the genome, respectively, which indicated that pangolin was the natural host of SARS-CoV-2 (Zhang T. et al., 2020). Nevertheless, further investigation is still needed to find the intermediate SARS-CoV-2 host.

To date, although the lethal rate of SARS-CoV-2 is currently lower than SARS-CoV, SARS-CoV-2 seems to be highly contagious according to the number of infections (Ashour et al., 2020). Furthermore, there are no specific drugs and/or vaccines to control the disease, and the epidemic of SARS-CoV-2 is posing a great threat for global public health. Accordingly, an in-depth study of SARS-CoV-2 will not only increase our understanding of the molecular mechanisms of its transmission, but also provide future potential treatment of comorbidities caused by SARS-CoV-2. In this review, we emphasize the genetic structure and pathogenic mechanism of SARS-CoV-2, clinical characterization of SARS-CoV-2 infection on different systems, including respiratory system, cardiovascular system, digestive system, and urinary system. Besides, potential treatments for SARS-CoV-2 are introduced, hoping to give practical advice on the treatment of SARS-CoV-2.

## Structure and Pathogenic Mechanism of Severe Acute Respiratory Syndrome Coronavirus-2

Since the current understanding of SARS-CoV-2 is still relatively small, clarifying its genetic structure and pathogenic mechanism may play an important role in its prevention and treatment.

### Genetic Structure of Severe Acute Respiratory Syndrome Coronavirus-2

**Figure 1:**
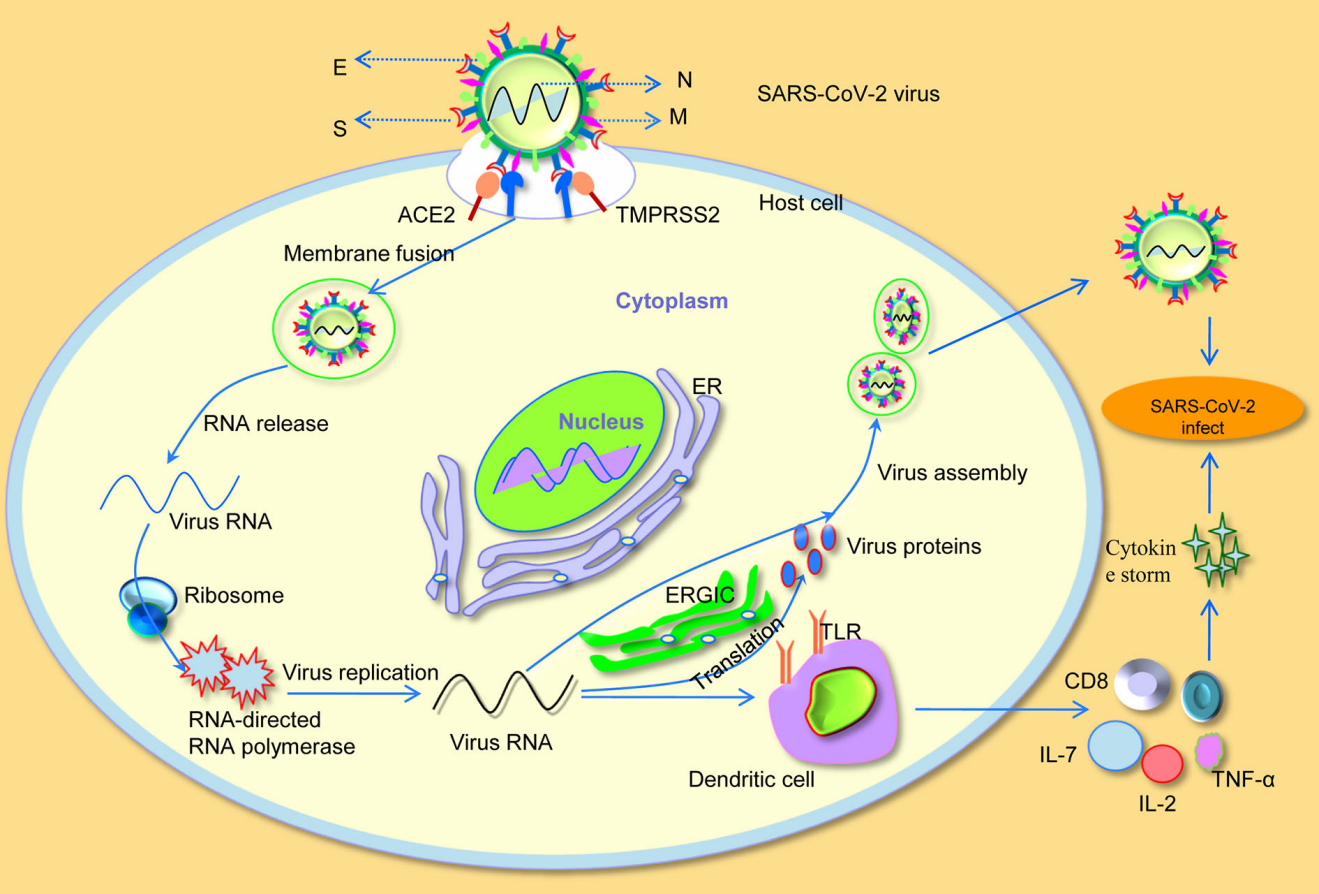
Severe acute respiratory syndrome coronavirus-2 (SARS-CoV-2) invades cellular mechanisms. S, spike; M, membrane; N, nucleocapsid; E, envelope; ER, endoplasmic reticulum; ERGIC, endoplasmic reticulum-Golgi intermediate compartment; IL-2, interleukin-2; IL-7, interleukin-7; TNF-α, tumor necrosis factor-α; TLR, toll-like receptor.

Although the origin of SARS-CoV-2 is still being investigated, the earliest genome-wide phylogenetic analysis data has implied that SARS-CoV-2 is composed of a single-stranded ribonucleic acid (RNA) structure, which belongs to the β-coronavirus (β-CoVs) and falls within the β-CoVs 2b lineage (Coutard et al., 2020; Zhai et al., 2020). Similar to other β-CoVs, SARS-CoV-2 virus particles look like a solar corona under transmission electron micrographs imaging. The virus particles are spherical and polymorphic, with a diameter of about 60 to 140 nm and specific spines of 9 to 12 nm in length (**Figure 1**) (Zhu N. et al., 2020). The viral genome of SARS-CoV-2 is about 29.8 kb, the G+C content is 38%, and there are six open reading frames common to coronaviruses and some other accessory genes. Moreover, SARS-CoV-2 has 5′ and 3′ terminal sequences, which are typical β-CoVs, 265 nt at 5′ ends and 358 nt at 3′ ends, respectively (Chan et al., 2020). SARS-CoV-2 contains four main structural proteins, namely spike (S), membrane (M), nucleocapsid (N), and envelope (E) proteins. Interestingly, the S, M, and E proteins are all embedded in the viral envelope, while the N protein is the only protein that interacts with the viral RNA at the core of the viral particle to form the nucleocapsid (Fehr and Perlman, 2015). According to previous reports, the severely glycosylated S protein forms a homotrimeric spikes on the surface of the virus particle, and mediates receptor binding to the host cell membrane through the receptor binding domain (RBD) of the S1 domain. Particularly, SARS-CoV-2 shares 89.8% sequence identity in S2 subunits with SARS-CoV, implying their similar mechanism of membrane fusion through S2 subunit (Xia et al., 2020). The M and E protein play a critical role in coordinating virus assembly and forming mature viral envelopes, while the N protein binds to the viral RNA and is involved in the transcription and replication of viral RNA, as well as packaging of the encapsidated genome into virions (Ashour et al., 2020; Nishiga et al., 2020).

### Pathogenic Mechanism of Severe Acute Respiratory Syndrome Coronavirus-2

Understanding the underlying pathogenic mechanism of SARS-CoV-2 is a key requirement for determining its effective treatment. Notably, a recent study has found that the binding affinity of SARS-CoV2 on the host cell for ACE2 receptor and RBD in S protein is more than 10 to 20 times higher than that of SARS-CoV, which may also be related to the increased infectivity and spread of SARS-CoV-2 (Wrapp et al., 2020). In addition, *in vitro* binding measurements also showed that the SARS-CoV-2 RBD binds to human ACE2 with an affinity of 15.2 nM, which was comparable to SARS-CoV spike protein with human ACE2, suggesting that RBD was a key functional component of the S1 subunit responsible for binding of SARS-CoV-2 to ACE2 (Tian et al., 2020). All the above studies indicate that SARS-CoV-2 effectively use human ACE2 as a cellular receptor, which could promote human-to-human transmission. Moreover, Markus et al. confirmed that SARS-CoV-2 not only used ACE2 for entry, but also required the cellular serine protease TMPRSS2 for S protein priming (Hoffmann et al., 2020). After binding to ACE2, the viral RNA genome was released into the cytoplasm. Once sufficient structural proteins and viral genomic RNA were formed, viral RNA forms virion-containing vesicles accompanied by envelope glycoproteins and nucleocapsid proteins, and then fused with the plasma membrane to release the virus (Sevajol et al., 2014). Particularly, viral assembly and budding occur in smooth-walled vesicles through a series of cooperative interactions that occur in the endoplasmic reticulum (ER) and endoplasmic reticulum Golgi intermediate compartment (ERGIC) (Masters, 2006).

In addition, the SARS-CoV-2 infected patients have higher plasma levels of cytokines such as interleukin-2 (IL-2), interleukin-7 (IL-7), and tumor necrosis factor-α (TNF-α) (Huang et al., 2020; Wang D. et al., 2020). More importantly, patients in critical conditions are often characterized by a reduction in peripheral blood T lymphocytes (Wang and Zhang, 2020). Apart from that, the number of CD4 and CD8 T cells in the peripheral blood of SARS-CoV-2 infected patients was significantly reduced, while their status was hyperactivated (Liu et al., 2020a; Xu Z. et al., 2020). All these findings suggest that, similar to SARS-CoV (Li et al., 2013), virus RNA of SARS-CoV-2 over-activates the host immune system by binding to toll-like receptor (TLR) on the surface of antigen-presenting dendritic cells, resulting in a cytokine storm and aggravating the infection of SARS-CoV-2 on host cells (**Figure 1**).

## Clinical Characteristics of Severe Acute Respiratory Syndrome Coronavirus-2

According to clinical reports, the typical common clinical manifestations of patients with SARS-CoV-2 infection are fever, cough, dyspnea, headache, pneumonia, and fatigue (Zhang J. J. et al., 2020; Zhou et al., 2020). In addition to respiratory symptoms, some patients also exhibit non-respiratory symptoms, diarrhea, heart failure, and acute kidney injury included, implying that SARS-CoV-2 can also invade other organs (Chen T. et al., 2020; Xu X. W. et al., 2020). Quantitative reverse transcriptase polymerase chain reaction (QRT-PCR) analysis of 72 human tissues shows that ACE2 is highly expressed in the bronchus, lung parenchyma, heart, kidney, and gastrointestinal tract (Harmer et al., 2002). More recently, through single-cell RNA-seq data analyses, Zou et al. identified that in addition to the above-mentioned organs, esophagus, and bladder also exhibit ACE2 expression (Zou et al., 2020). As mentioned previously, the SARS-CoV-2 is similar to SARS-CoV, invading human cells through the ACE2 receptor. Hence, in respiratory system, cardiovascular system, digestive system, and urinary system, organs related to ACE2 expression may act as targets and thus are susceptible to SARS-CoV-2 infection.

### Respiratory System Injury

The SARS-CoV-2 first primarily infects the lower airway and binds to ACE2 on pulmonary capillary endothelial cells (ECs), thus increasing the viral load in the lungs. Subsequently, most patients with SARS-CoV-2 often show dyspnea, acute respiratory failure, ARDS, etc. (Liu J. et al., 2020). Meanwhile, in severe cases, bilateral lung involvement with ground glass opacification is a more common chest syndrome on computed tomography scans (Schultz, 2020). In clinical autopsy, histological examination of the virus showed bilateral diffuse alveolar damage with cellular fibromyxoid exudates (Xu Z. et al., 2020). Moreover, the SARS-CoV-2 viral load detected in the patient respiratory tracts is positively correlated with the severity of lung disease, and total lymphocytes, such as CD4+ T cells, CD8+ T cells as well as B cells, and natural killer (NK) cells decreased in SARS-CoV-2 patients (Liu Y. et al., 2020; Wang F. et al., 2020). This indicates that SARS-CoV-2 may take part in a pro-inflammatory cytokine response through activating immune cells, inducing the secretion of inflammatory cytokines and chemokines to pulmonary vascular ECs (Jiang et al., 2020).

**Figure 2:**
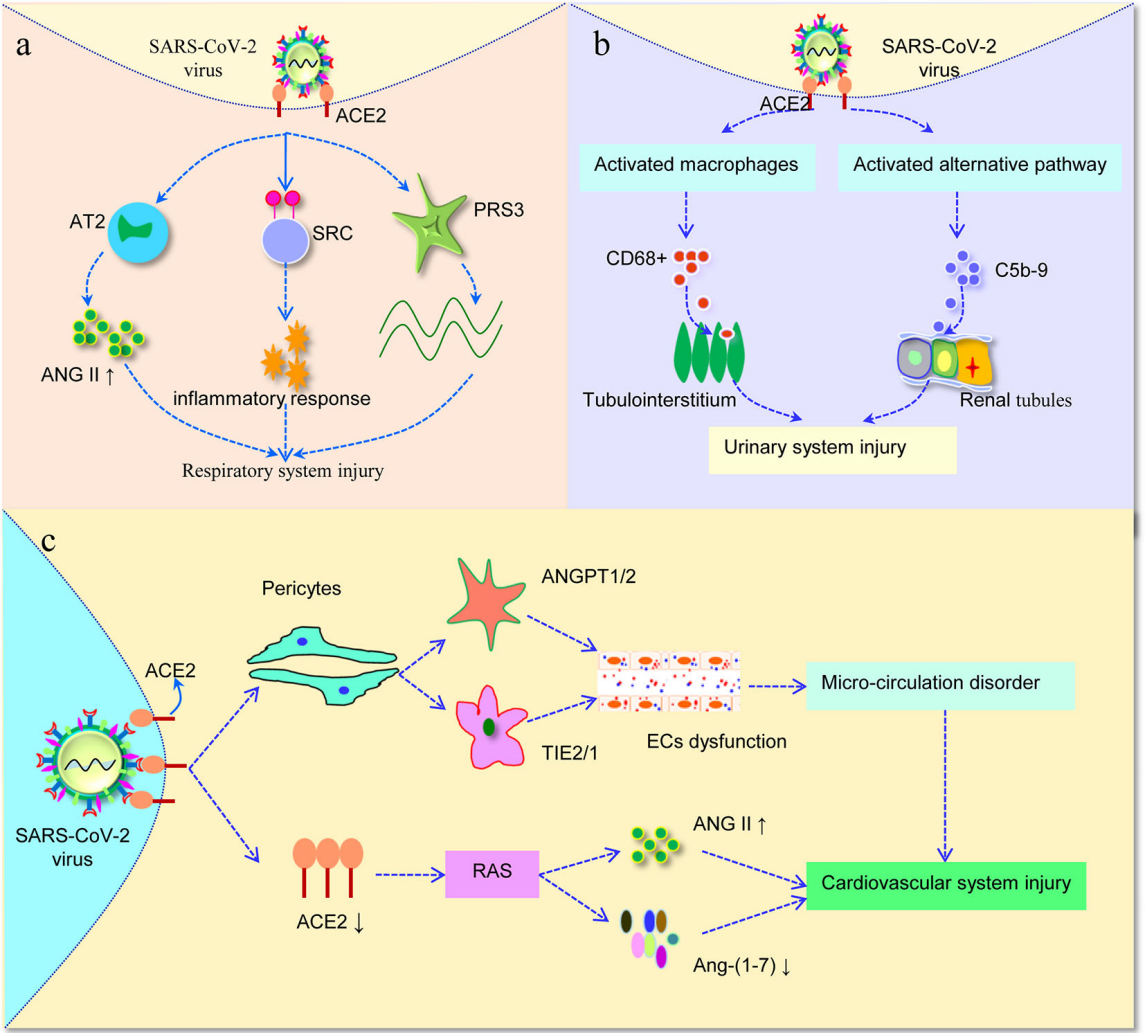
Severe acute respiratory syndrome coronavirus-2 (SARS-CoV-2) invades different systems. **(A)** SARS-CoV-2 bind to ACE2 in the lung, further affects the level of ANG II. Furthermore, the increased expression of ACE2 affected RPS3 and SRC, the two hub genes involved in viral replication and inﬂammatory responses. **(B)** SARS-CoV-2 virus infection not only induces CD68+ macrophages inﬁltrated into tubulointerstitium, but also enhances complement C5b-9 deposition on tubules, leading to urinary system injury. **(C)** Once SARS-CoV-2 infection attack pericytes in the human heart, then through balance between ANGPT1/2 and TIE2/1 result in capillary ECs dysfunction, thus inducing micro-circulation disorder. In addition, after binding of SARS-CoV-2 with ACE2, it is likely to alter the function of RAS, and then cause cardiovascular damage. AT2, angiotensin II type 2 receptor; ANG II, angiotensin II; RAS, renin-angiotensin system; ECs, endothelial cells.

Previous study has shown that SARS-CoV can bind to its receptor ACE2, resulting in increased angiotensin II (ANG II) level in mouse blood samples and induced acute lung injury, *via* angiotensin II type 2 receptor (AT2) (Imai et al., 2005). Interestingly, in a report concerning 12 SARS-CoV-2 infected patients, plasma ANG II levels were significantly increased and were linearly correlated with viral load and lung injury (Liu Y. et al., 2020). This may explain why acute lung injury is observed in some SARS-CoV-2-infected patients (**Figure 2A**). In addition, Li et al. used bioinformatics to identify the expression characteristics and possible regulatory networks of ACE2 in the lungs. The calculations supported the hypothesis that the replication and assembly of SARS-CoV-2 was facilitated by such process: the transcriptome of epithelial cells changes after SARS-CoV-2 infection, which increases the expression of ACE2, further affecting ribosomal proteins RPS3 and cytokine secretion associated proteins SRC that play key roles in viral replication and the inflammatory response (Li G. et al., 2020).

### Digestive System Injury

While ACE2 is highly expressed in the lungs, it is also abundantly expressed in the digestive system, especially in esophageal epithelial cells and the absorptive enterocytes from ileum and colon, providing a prerequisite for SARS-CoV-2 infection (Xu H. et al., 2020). Gastrointestinal (GI) symptoms such as diarrhea, vomiting, and nausea have been reported in 2 to 10% of patients with SARS-CoV-2 infected (Yeo et al., 2020). Besides, SARS-CoV-2 RNA can be detected in the stomach, duodenum, and rectum of severe patients (Lin et al., 2020). Recently, the SARS-CoV-2 virus was found in the stool of the patient, which directly proved that SARS-CoV-2 can be transmitted through the stool (Xiao F. et al., 2020). Therefore, it was suggested that the GI might be a potential transmission route and target organ for SARS-CoV-2. Although there is no significant damage in the mucosal epithelial of esophagus, stomach, duodenum, and colorectum judged by H&E staining, a great number of plasma cells infiltrated by SARS-CoV-2 and lymphocytes with interstitial edema in stomach, duodenum, and rectum lamina propria were found histologically from 73 hospitalized patients infected with SARS-CoV-2 (Xiao F. et al., 2020). In addition to GI symptoms, SARS-CoV-2 infected patients may have liver damage with elevated level of enzymes found in blood tests. Recent studies on SARS-CoV-2 have shown that the incidence of liver injury is 14.8 to 67.2%, which is mainly manifested by abnormal ALT/AST levels and slightly elevated bilirubin levels (Lin et al., 2020; Phipps et al., 2020; Xu L. et al., 2020). However, in a retrospective analysis of 115 patients from China, the main serum indicators of liver function such as ALT, AST, GGT, and LDH in SARS-CoV-2 patients were not significantly different from the control group, indicating that liver was not the main target organ of SARS-CoV-2 infection (Zhang Y. et al., 2020), which was consistent with previous studies reporting that the ACE2 was weakly expressed in human hepatocytes. In fact, a retrospective study reported that the abnormal liver function in patients with SARS-CoV-2 might be caused by lopinavir or litonavir used as antivirals to treat SARS-CoV-2 infection (Fan et al., 2020). Thus, these drugs with digestive system damage should be given with caution.

Currently, there is limited research on the mechanisms of SARS-CoV-2-related digestive system. Of note, online datasets and bioinformatics methods showed that the expressions of messenger RNA (mRNA) and protein of the ACE2 gene were highly expressed in human small intestinal epithelial cells, and then the SARS-CoV-2 virus might enter the small intestinal epithelial cells through binding to ACE2, which induced GI symptoms and inflammation susceptibility (Zhang H. et al., 2020).

### Urinary System Injury

Lung lesions are considered to be the main major damage caused by SARS-CoV-2 infection. However, kidney injury has also been reported to occur in mild or severe cases during the course of the disease. Single-cell RNA sequencing (scRNA-seq) analysis was performed to identify candidate kidney host cells, and ACE2 gene had been shown relatively high expression in podocytes, proximal straight tubule cells, and bladder urothelial cells. Parallelly, comparative analysis showed that the expression of ACE2 gene in the kidney cells was no less than that in the lung, esophagus, and small intestine (Pan et al., 2020; Zou et al., 2020). Moreover, recent human tissue RNA-sequencing data indicated that ACE2 expression in kidney was nearly 100 times higher than that in the lung, which suggested that the kidney was more vulnerable to SARS-CoV-2 infection (Cheng et al., 2020). An early research data showed that the kidney injury was the second most common organ injury caused by SARS-CoV-2. In particular, kidney injury was more severe in critical ill patients than in severe patients (Li Y. et al., 2020). In another multi-centered, retrospective, observational study including 193 SARS-CoV-2-infected patients, CT data exhibited radiographic abnormalities of kidney. Beyond that, univariate Cox regression analyses revealed that the death of SARS-CoV-2-infected patients was significantly associated with the elevated proteinuria, hematuria, blood in urine (BUN), and Scr, respectively, which suggested that the general presence of kidney dysfunctions in SARS-CoV-2-infected patients (Li Z. et al., 2020). Subsequently, Diao et al. analyzed renal tissue morphology from autopsies, and HE staining demonstrated severe acute tubular necrosis and lymphocyte infiltration in kidney tissues (Diao et al., 2020). Furthermore, a recent study reported that SARS-CoV-2 virus particles had successfully been detected from the urine of SARS-CoV-2-infected patients (Guan and Zhong, 2020). Collectively, considering that SARS-CoV-2 RNA has been detected in the kidney and urine of patients, it is possible that the urinary system is also a site of viral infection and replication.

To further analyze how SARS-CoV-2 affected renal function, six patients with SARS-CoV-2 postmortem examinations were analyzed by immunohistochemistry. As a result, SARS-CoV-2 infection not only induced high levels of CD68+ macrophages to infiltrated into tubulointerstitium, but also initiated complement C5b-9 assembly and deposition on renal tubules, suggesting that proinflammatory cytokines derived from macrophages and triggering C5b-9 deposition would induce tubular damage and consequently lead to acute renal tubular injury (**Figure 2B**) (Diao et al., 2020). However, the underlying mechanism of kidney involvement in patients infected with SARS-CoV-2 is unclear, and further research is still urgently needed for fully understand the effect of SARS-CoV-2 on the kidney.

### Cardiovascular System Injury

Although SARS-CoV-2 is characterized by fever and respiratory manifestations, recent research data show that concurrent cardiovascular symptoms are not uncommon, and often present as acute heart injury, accompanied by cardiomyopathy, ventricular arrhythmias, and hemodynamic instability (Hendren et al., 2020). Moreover, it has been reported that the blood coagulation function of SARS-CoV-2-infected patients seems markedly abnormal compared with healthy controls. Specially, D-dimer, fibrinogen degradation products, and fibrinogen values were found to be significantly increased, while anti-thrombin was found to be significantly lower (Han et al., 2020). Previous studies have confirmed that the ACE2 is widely expressed in cardiomyocytes, cardiac fibroblasts, and coronary ECs, which are also a regulator for heart function (Guo et al., 2020). Remarkably, pericyte, a type of perivascular mural cells, has been proved to be associated with high expression of ACE2 and specifically expressed, suggesting that pericyte may act as a potential targeted host cell for SARS-CoV-2 virus in the human heart (Chen et al., 2020a). In a retrospective multicenter study, the factors associated with mortality of 68 death patients and 82 discharged patients with laboratory-confirmed SARS-CoV-2 infection were evaluated. It should be noted that compared to survived patients, cardiovascular disease is linked to a significantly increased risk of death in patients who have died (p < 0.001) (Ruan et al., 2020). In parallel, dead cases had the higher levels of myoglobin and cardiac troponin, thus confirming relevant findings (Li J. W. et al., 2020). Meanwhile, degeneration and necrosis of cardiomyocytes was found through sporadic autopsy and biopsy histopathology, and a few of monocytes, lymphocytes, and neutrophils infiltration could be seen in the cardiac myocyte (Yao et al., 2020). As special populations, children are thought to be less susceptible than adults to SARS-CoV-2. Albeit uncommon, however, it has been reported that children infected with SARS-CoV-2 might develop into a highly inflammatory shock with features akin to Kawasaki disease (Riphagen et al., 2020). For instance, an Italian study reported that there was 30-fold increased incidence of Kawasaki-like disease during the SARS-CoV-2 epidemic (Verdoni et al., 2020).

Apart from the acute heart injury, another distinctive feature noted in severe SARS-CoV-2-infected patients was commonly accompanied by coagulopathy or vasculopathy, including disseminated intravascular coagulation, venous and arterial thromboembolic diseases, and the feature was associated with poor prognosis (Bikdeli et al., 2020; Goldman et al., 2020). In a study of 184 patients with severe SARS-CoV-2-infected from 3 hospitals in the Netherlands, Klok et al. (2020) reported that with the exacerbation and progress of the disease, 31% cases suffered from thrombotic complications, among them, venous thromboembolism (VTE) accounts for 27% and arterial thrombotic events for 3.7%. Similarly, 80 consecutive full autopsies of SARS-CoV-2-infected patients were observed by Edler *et al*., the corresponding results presented that the prevalence rate of VTE was as high as 40% (Edler et al., 2020). These findings suggest that VTE is more common than arterial thromboembolism in SARS-CoV-2-infected patients. To date, there are several ways in which the SARS-CoV-2 pandemic may affect the thrombosis events, including hyper-inflammatory processes, diffuse intravascular coagulation, platelet activation, severe hypoxemia, and ECs dysfunction (de Carranza et al., 2020; Iba et al., 2020). However, given the pathogenesis of thrombosis events is multifactorial and has not been fully understood, further research is required to elucidate the molecular basis of thrombosis and pathophysiological mechanisms in SARS-CoV-2-infected patients.

To further illuminate the potential mechanism of myocardial injury, Chen et al. (2020a) analyzed cell-cell interaction analysis with pericytes, finding that ECs showed extensive linkages with pericytes through balance between ANGPT1/2 and TIE2/1. It means that once SARS-CoV-2 attack pericytes in the human heart, capillary ECs dysfunction occurs, thus inducing micro-circulation disorder. In addition, ACE2, a key enzymatic component of the renin-angiotensin system (RAS), is likely to alter the function of RAS after binding with SARS-CoV2. Since the loss of ACE2 may transform the RAS system by inducing an overall higher level of ANG II, a peptide with multiple functions that promote cardiovascular, and produce lower amount of Ang-(1–7) that antagonizes ANG II (**Figure 2C**) (Alifano et al., 2020; South et al., 2020). Nevertheless, it is yet unknown whether the observed cardiovascular damage is due to SARS-CoV-2 virus injury or RAS response that affects the secretion of ANG II/Ang-(1-7). Therefore, more evidence from laboratory and clinical research is needed.

Previous studies have reported that SARS-infected male patients show wide-spread germ cell destruction with little or no sperm in the seminiferous tubules (Xu et al., 2006). However, none were found to be positive for SARS-CoV-2 by RT-PCR assay in semen or vaginal fluid (Paoli et al., 2020; Qiu L. et al., 2020), suggesting that the likelihood might be low for transmitting SARS-CoV-2 to sexual partners through semen or vaginal fluids. In addition, while the neurological manifestations of SARS-CoV-2 have not been properly studied, it is likely that a growing number of SARS-CoV-2 patients, especially those who suffer from severe epilepsy, have disturbed consciousness and viral encephalitis (Wu Y. et al., 2020). Taken together, these results demonstrate that the SARS-CoV-2 can cause multiple system injury. Therefore, it is of great urgency to develop drugs and vaccines to treat SARS-CoV-2 virus.

## Potential Treatments for Coronavirus Disease 2019

Currently, the epidemic of SARS-CoV-2 is still posing a great threat for global public health, and numerous trials have been conducted around the world to test potential treatment and preventative options. Noticeably, vaccines have proven to be the most effective and economical means of preventing and controlling infectious diseases. Up to now, potential fragments of SARS-CoV-2 as antigens in vaccine development include the RBD domain, the S protein, the whole cell antigens, and the N-terminal domains, etc. (Tai et al., 2020; Zhang J. et al., 2020). Particularly, the S protein can be directly recognized by host immune system, and mediates viral entry into host cells by first binding to the receptor ACE2, which is crucial for the subsequent virus to enter the target cells and cause subsequent pathogenicity (Marohn and Barry, 2013; Wrapp et al., 2020), making it the most promising antigen formulation. In a dose-escalation, single-center, non-random phase 1 trial, an Ad5-vectored COVID-19 vaccine was tested among healthy adults in China. This first trial in humans showed that no serious adverse event was noted in 28 days after vaccination, ELISA antibodies and neutralizing antibodies increased significantly on day 14 and peaked 28 days after vaccination, as well as specific T-cell response peaked on day 14 after inoculation (Zhu F. C. et al., 2020). However, generally speaking, the development of new vaccines needs 10 to 20 years, and the success rate is less than 10%, even vaccines enters clinical trials (Zhang J. et al., 2020).

In addition, it should be noted that there is no treatment that has been proven to be effective for critically ill patients, and only supportive measures can be applied including treatment with antiviral drugs, corticosteroids, and noninvasive mechanical ventilation, etc. Unfortunately, while antiviral drugs such as chloroquine, lopinavir, and Lianhuaqingwen have been reported to be potentially effective, supportive clinical data are not available for all (Liu S. et al., 2020; Runfeng et al., 2020). For instance, in a large multinational real-world analysis, Mandeep et al. did not observe any benefit of hydroxychloroquine or chloroquine on in-hospital outcomes early after diagnosis of COVID-19 (Mehra et al., 2020). Furthermore, considering that corticosteroids may delay the elimination of the virus and increase the risk of secondary infections, especially for patients with compromised immune systems, the WHO guidance on the clinical management of SARS-CoV-2 doesn’t recommend to use corticosteroids during treatment (Isidori et al., 2020). However, in a controlled, open-label randomized evaluation of therapy trial of dexamethasone in hospitalized patients with SARS-CoV-2, Horby et al. found that among patients receiving invasive mechanical ventilation, the mortality rate of the dexamethasone group was lower than that of the usual care group (29.3 *vs.* 41.4%; rate ratio, 0.64; 95% CI, 0.51 to 0.81) (Horby et al., 2020). Thus, it is of great value to develop new treatments targeting SARS-CoV-2 to save COVID-19 patients. To solve this problem, more and more research is developing new treatments for SARS-CoV-2.

### Anticoagulant and Antithrombotic Agents

As described above, SARS-CoV-2 may predispose to venous and arterial thromboembolism. Hence, given the high risk of thrombotic events in SARS-CoV-2-infected patients, appropriate anticoagulation prophylaxis and thromboembolism imaging check in each symptomatic patient affected by SARS-CoV-2 have gained tremendous attention over the last months. Recently, the WHO and the International Society on Thrombosis and Haemostasis have recommended prophylactic dose low molecular weight heparin (LMWH), which should be considered in all hospitalized patients with SARS-CoV-2 (Bikdeli et al., 2020; Thachil et al., 2020). In fact, it is important to note the multiple roles of LMWH, on the one hand, as preventer of the thromboembolic process and thrombotic complications, on the other hand, as a powerful inhibitor of anti-viral and anti-inflammatory (Kipshidze et al., 2020; Talasaz et al., 2020). Additionally, accumulating evidence indicated that prophylactic anticoagulation could reduce the risk of VTE and mortality in severely ill patients. For example, in a retrospective study involving 2,773 SARS-CoV-2-infected patients of whom only 28% received anticoagulant therapy, the hospital mortality was lower in patients received systemic anticoagulation (29.1%) compared to mortality in patients not treated with anticoagulation (62.7%) (Paranjpe et al., 2020). More remarkably, several groups have reported that VTE may occur despite standard thromboprophylaxis (Langer et al., 2020). Therefore, more prospective and randomized trials are needed to validate the protective effect of anticoagulation therapy in the treatment of SARS-CoV-2-infected patients.

### Monoclonal Antibody (MAb)

MAb therapy is a new breakthrough in the field of infectious disease prevention, which can overcome many shortcomings associated with serum therapy or intravenous immunoglobulin preparations, such as specificity, purity, and safety (Shanmugaraj et al., 2020). Thus, MAb therapy has been widely used in the treatment of various diseases. In one clinical trial, Xu X. et al. (2020) aimed to assess the efficacy of IL-6 receptor-targeted MAb tocilizumab in severe patients with SARS-CoV-2. Preliminary data showed that tocilizumab could immediately improve the clinical outcomes of severe and critical patients, which was an effective treatment for reducing mortality. However, more randomized clinical trials are needed to further evaluate the safety and efficacy of tocilizumab in treating patients with SARS-CoV-2. Furthermore, one SARS-CoV-specific human MAb, CR3022, was found to bind potently with SARS-CoV-2 RBD in a KD of 6.3 nM, without showing any competition with ACE2 for the binding to SARS-CoV-2 RBD. All these results imply that CR3022 may be a drug candidate for the prevention and treatment of SARS-CoV-2 infections (Tian et al., 2020). Notably, previous evidence has proven that convalescent plasma from patients who have recovered from SARS-CoV-2 infection can significantly reduce the relative risk of mortality of patients without the occurrence of severe adverse events (Bloch et al., 2020; Chen et al., 2020b). For instance, a case series of four critically ill patients with SARS-CoV-2 infection in China reported improvement in clinical symptoms after transfusion with convalescent plasma as evidenced by stopping mechanical ventilation, reducing in viral loads, improving oxygenation index and P_O2_ (Zhang B. et al., 2020). Therefore, it is worthwhile to develop anti-viral MAb using antibodies from convalescent SARS-CoV-2-infected patients.

### Mesenchymal Stem Cell (MSC)

MSC have anti-inflammatory functions, which can reduce the occurrence of cytokine storm syndrome and ARDS (Xiao K. et al., 2020). At the same time, MSC can secrete a variety of cytokines through paracrine, or directly interact with immune cells such as T cells, B cells, dendritic cells, macrophages, and NK cells to produce immuneomodulation (Atluri et al., 2020). As mentioned, severe SARS-CoV-2 patients are characterized by pneumonia, lymphopenia, and a cytokine storm (Golchin et al., 2020). Accordingly, it is worth a bold attempt to use MSC transplantation to treat severe SARS-CoV-2 to curb the progression of critically infected patients. Presently, there is no approved MSC-based method for the treatment of SARS-CoV-2 patients, but a growing number of clinical investigations are on being conducted. According to the investigation of Leng et al. (2020), seven SARS-CoV-2 patients showed a greatly improved pulmonary function and alleviated symptoms after MSC transplantation. Moreover, by transplanting MSC in 17 patients with H7N9 induced ARDS, Chen et al. found that MSC transplantation significantly lower the mortality compared with control group. Additionally, there were no adverse effects on human body after MSC administration during the 5 years follow-up. Because H7N9 and the SARS-CoV-2 share similar complications such as ARDS, lung failure, and fulminant pneumonia, MSC therapy can be a possible alternative for treating SARS-CoV-2 (Chen J. et al., 2020). Even more interesting, MSCs are ACE2 negative and thus, not infected by the H7N9 virus (Bari et al., 2020). Therefore, Atluri et al. suggested that MSC-based treatment might be an ideal candidate for clinical trials, or at least a combination of treatment for SARS-CoV-2 patients (Atluri et al., 2020). However, more research is needed in a larger patient cohort to further evaluate the safety and efficacy of MSCs in the treatment of SARS-CoV-2.

### Bioinformatics and Artificial Intelligence

In general, the experimental studies to reveal the molecular mechanisms behind the viral infections and the design of vaccine or antiviral drugs are costly and require years to develop. However, bioinformatics can predict and discover potential drug, targets, and side effects by analyzing and mining published large-scale multi-group within only a few months. As such, bioinformatics has been widely used in drug development and screening of SARS-CoV-2. For example, to provide a comprehensive structural genomics and interactive analysis of SARS-CoV-2, Dmitry et al. used integrated bioinformatics to reveal the evolutionary conservation and divergence of functional regions of SARS-CoV-2 as well as make a comprehensive homology modeling analysis of the 3D structure of SARS-CoV-2 proteins, which provided a protein structure reference for the drug design of SARS-CoV-2 (Wang X. et al., 2020). Similarly, Salman et al. identified the chymotrypsin-like protease inhibitors of SARS-CoV-2 from the Food and Drug Administration (FDA) approved antiviral drugs and their internal database by computational drug design methods. As a result, five synthetic and natural compounds were identified as promising hits including remdesivir, saquinavir, darunavir, Syn-16, and Nat-1 (Khan et al., 2020). Besides, the Lancet has recently published a study using Benevolent artificial intelligence to screen out baricitinib as potentially effective agent against SARS-CoV-2 infection (Richardson et al., 2020). Moreover, Ethan et al. combined structural biology and machine learning to identify SARS-CoV-2 T-cell and B-cell epitopes. They found 405 likely T-cell epitopes with strong MHC-I and MHC-II presentation scores, and 2 potential neutralizing B-Cell epitopes on S protein, which could be used to develop more effective vaccines and recognize neutralizing antibodies (Chen Y. L. et al., 2020).

In particular, the effectiveness of Chinese medicine treatment in controlling infectious diseases was demonstrated during the outbreak of SARS in 2003 (Liu et al., 2012). Through molecular docking and network pharmacology analysis, Zhang et al. screened out 26 Chinese herbal medicines with a high probability, which might be directly inhibiting SARS-CoV-2 (Zhang D. H. et al., 2020). Nevertheless, clinical studies on the treatment of COVID-19 with traditional Chinese medicine are rarely published. Apart from the above measures, recently, an effective way to deal with the possible mutation of the virus attracted extensive attention. It has been found that adeno-associated virus can be used as vectors to deliver CRISPR/Cas13d gene editing system to edit and chew up SARS-CoV-2 and other RNA virus, which may be a new method for treatment and prevention of multiple RNA virus infections (Nguyen et al., 2020). Overall, the above potential treatments strategies are based on the latest research data for SARS-CoV-2, and more clinical validation is needed.

## Conclusion and Future Perspectives

At present, although epidemic prevention and control efforts have achieved remarkable results in China, SARS-CoV-2 is becoming a global pandemic, and there are still many problems that need to be resolved. On the one hand, it had been well recognized that SARS-CoV-2 invaded the cells of human *via* binding to ACE2 receptors and contributed to the disorder of digestive, cardiovascular, and urinary systems. However, there are still some deficiencies in our understanding of SARS-CoV-2. On the other hand, there is no standard drug regimen or vaccine available against COVID-19 at present, and developing and identifying effective anti-SARS-CoV-2 interventions remains a major challenge. Thus, it is important to clarify the pathogenesis, the overall picture, and the course characteristics of SARS-CoV-2, which can provide novel insights to explore the medicine or vaccine. Moreover, and remarkably, a study reported that there were four patients in China, who met criteria for discharge or quarantine cessation, still had positive RT-PCR test after recovery, which caused widespread concern (Lan et al., 2020). More recently, Xiao et al. reported that in a study of 70 patients with COVID-19, 21.4% of the patients tested positive for SARS-CoV-2 by RT-PCR after two consecutive negative results (Cao et al., 2020). These findings suggest that at least some of the recovered patients still may be virus carriers, and current criteria for hospital discharge of quarantine and continued patient management may need to be re-evaluated. Currently, with major domestic efforts and international support, the epidemic in China is being gradually controlled and remarkable results have been achieved. However, the global epidemic has now become a pandemic. Therefore, in order to reduce infection and mortality and effectively contain the epidemic, countries need to strengthen cooperation, share experience and data, and carry out targeted intervention and treatment of the epidemic.

## Author Contributions

CZ and BS wrote the first draft of the manuscript. XZ and BZ revised the manuscript. All authors contributed to the article and approved the submitted version.

## Conflict of Interest

The authors declare that the research was conducted in the absence of any commercial or financial relationships that could be construed as a potential conflict of interest.
